# Supplementary material for: Duckweeds for Phytoremediation of Polluted Water
Source: Plants (Basel). 2023 Jan 29;12(3):589. doi: 10.3390/plants12030589 (PMC9919746; doi:10.3390/plants12030589)
Supplement: Supplementary file 1 [file plants-12-00589-s001.zip › plants-2058988-Table S1.pdf]

Table S1. Studies of heavy metals uptake by duckweeds				
**Duckweed species**	**Metal(s)**	**Initial concentration (mg/L)**	**Removal rate**	**Reference**
Lemna gibba	Cu, Zn	0.001, 0.1	70-80%	[169]
	Cd		～90%	
	Pb	2, 4, 10, 15	91.0-96.4%	[118]
	Cr		86.2-94.8%	
	Pb	---[a]	60%	[170]
	Ni		60%	
	Mn		62%	
	Cu		57%	
	Cr	1-9	up to 98.6%	[171]
	Cd		up to 94.6%	
	Cu	0.1	80%	[172]
		0.5	60%	
	Ni	0.1	15%	
		0.3	10%	
		0.5	10%	
	Pb	10	98.1	[122]
		2	60.1	
	Cd	10	41.6	
		2	84.8	
Lemna minor	Cu	0.5	99.8%	[173]
	Hg	0.25	99.53%	
	Pb		97.88%	
	Zn		94.37%	
	Pb	---[b]	62%	[170]
	Ni		68%	
	Mn		77%	
	Cu		58%	
	As	0.5	70%	[154]
	Cd	0.5-3	42-78%	[119]
	Cd	0.038	95	[123]
		0.054	94.5	
	Cu	0.062	94.7	
		0.032	92.5	
	Pb	0.608	97.6	
	Ni	0.059	99.9	
		0.419	90	
	Cr	0.1	94	[116]
	Pb	0.25	36	
	Cd	1.5	33	
	Cu	0.05	29	
	Zn	1.25	51-82	
	Cd	0.11	95	[174]
	Pb	0.21	93	
	Zn	0.065	81.2	
	Cu	0.064	86.5	
	Pb	10	99.99	[175]
	Ni		99.3	
	Pb	16[c]	98.7	[176]
	Cu	12[c]	99.8	
	Zn	43[c]	72	
	Cd	5.1[c]	99.6	
	Zn	1.47	83	[177]
	Pb	0.83	78	
	Fe	1.17	77	
	Cu	0.69	91	
	Ni	1.21	76	
	Zn	1-20	40-83	[178]
Lemna minuta	Zn	1-20	35-89	
Lemna trisulca	Zn	1-20	49-97	

Spirodela polyrhiza	Zn	1-5	>90%	[179]
	Cu			
	Cr			
	Fe			
	Cd			
	Cd	0.5-3	52-75%	[119]
	Pb	1	53	[180]
	Cd	1	53	
	Cu	65	79	[181] (waste water sample from India)
	Pb	26	95	
	Zn	212	66	
	Cr	118	53	
	Co	7.2	28	
	Mn	8	20	
	Hg	23	45	
	Ni	19.3	9	
	Cu	63	74	[181] (waste water sample from Kerala)
	Pb	34.4	91	
	Zn	301	62.4	
	Cr	121	49	
	Co	8	40	
	Mn	7.3	30.1	
	Hg	3.4	53	
	Ni	22.3	22	
	Fe	5.3	98.1	
	Cd	3	100	
a, waste water in circulation; b, waste water in circulation; c, concentration unit is μg/L				

References:

169. Megateli, S.; Semsari, S.; Couderchet, M. Toxicity and removal of heavy metals (cadmium, copper, and zinc) by *Lemna gibba*. *Ecotoxicology and Environmental Safety*, **2009**, 72(6), 1774-1780
170. Yilmaz, D. D.; Akbulut, H. Effect of circulation on wastewater treatment by *Lemna gibba* and *Lemna minor* (Floating Aquatic Macrophytes). *International Journal of Phytoremediation*, **2011**, 13(10), 970-984.
171. Ekta, C.; Praveen, S. Chromium and cadmium removal from wastewater using duckweed-*Lemna gibba* L. and ultrastructural deformation due to metal toxicity. *International Journal of Phytoremediation*, **2019**, 21(3), 279-286.
172. Khellaf, N.; Zerdaoui, M. Growth response of the duckweed *Lemna gibba* L. to copper and nickel phytoaccumulation. *Ecotoxicology (London,England)*, **2010**, 19(8), 1363-1368.
173. Romero-Hernández, J.A.; Amaya-Chávez, A.; Balderas-Hernández, P.; Roa-Morales, G.; González-Rivas, N. Balderas-Plata, M.A. Tolerance and hyperaccumulation of a mixture of heavy metals (Cu, Pb, Hg, and Zn) by four aquatic macrophytes. *International Journal of Phytoremediation,* **2017**, 19(3), 239-245.
174. Basile, A.; Sorbo, S.; Conte, B.; Cobianchi, R.C.; Trinchella, F.; Capasso, C.; Carginale, V. Toxicity, accumulation, and removal of heavy metals by three aquatic macrophytes, *Int. J. Phytoremediat.* **2012**, 14, 374–387.
175. Kaur, L.; Gadgil, K.; Sharma, S. Role of pH in the accumulation of lead and nickel by common duckweed (*Lemna minor*). *International Journal of Bioassays* **2012**, 01, 12, 191-195.
176. Azeez, N.M.; Sabbar, A.A. Efficiency of duckweed (*Lemna minor L.*) in phytotreatment of wastewater pollutants from Basrah oil refinery. *Journal of Applied Phytotechnology in Environmental Sanitation* **2012**, 1, 4, 163-172.
177. Daud, M.K.; Ali, S.; Abbas, Z.; Zaheer, I.E.; Riaz, M.A.; Malik, A.; Hussain, A.; Rizwan, M.; Rehman, M.Z.; Zhu, S.J. Potential of duckweed (*Lemna minor*) for the phytoremediation of landfill leachate. *Journal of Chemistry* 2018, 2018, 1-9. https://doi.org/10.1155/2018/3951540

178. Jafari, N.; Akhavan, M. Effect of pH and heavy metal concentration on phytoaccumulation of zinc by three duckweed species. *American–Eurasian Journal of Agricultural and Environmental Science* 2011, 10(1), 34-41.
179. Kumar, M.V.; Tripathi, B.D. Concurrent removal and accumulation of heavy metals by the three aquatic macrophytes. *Bioresource Technology*, **2008**, 99(15), 7091-7097, doi:10.1016/j.biortech.2008.01.002
180. Al-Balawna, Z.A. Remove heavy metals (Cd and Pb) from irrigation water in Jordan valley by using duckweeds (*Spirodela polyrhiza*). *Acta Scientific Agriculture* **2020**, 4(2), 1-4, doi:10.31080/ASAG.2020.04.REMOVE-HEAVY-METALS-CD-AND-PB-FROM-IRRIGATION-WATER-IN-JORDAN-VALLEY-BY-USING-
181. Loveson, A.; Sivalingam, R.; Syamkumar, R. Aquatic macrophyte *Spirodela polyrrhiza* as a phytoremediation tool in polluted wetland water from Eloor, Ernakulam district, Kerala. *IOSR Journal of Environmental Science, Toxicology and Food Technology*. **2013**, 5(1), 51-58, doi:10.9790/2402-0515158
